# Persistent anxiety among high school students: Survey results from the second year of the COVID pandemic

**DOI:** 10.1371/journal.pone.0275292

**Published:** 2022-09-30

**Authors:** Olivia Yin, Nadia Parikka, Amy Ma, Philip Kreniske, Claude A. Mellins

**Affiliations:** 1 Irvington High School, Irvington, New York, United States of America; 2 HIV Center for Clinical and Behavioral Studies, NY State Psychiatric Institute and Columbia University, New York, New York, United States of America; King Khalid University, SAUDI ARABIA

## Abstract

**Introduction:**

National mental health surveys have demonstrated increased stress and depressive symptoms among high-school students during the first year of the COVID-19 pandemic, but objective measures of anxiety after the first year of the pandemic are lacking.

**Methods:**

A 25-question survey including demographics, the Generalized Anxiety Disorder-7 scale (GAD-7) a validated self-administered tool to evaluate anxiety severity, and questions on achievement goals and future aspirations was designed by investigators. Over a 2-month period, all students from grade 9–12 in a single high-school (n = 546) were invited to complete an online survey after electronic parental consent and student assent. Bi-variate and chi-square analyses examined demographic differences in anxiety scores and the impact on outcomes; qualitative analyses examined related themes from open-ended questions.

**Results:**

In total, 155/546 (28%) completed the survey. Among students with binary gender classifications, 54/149 (36%) had GAD-7 scores in the moderate or severe anxiety range (scores≥10), with a greater proportion among females than males (47% vs 21%, P<0.001). Compared to students with GAD-7<10, those with ≥ 10 were more likely to strongly agree that the pandemic changed them significantly (51% vs 28%, p = 0.05), made them mature faster (44% vs 16%, p = 0.004), and affected their personal growth negatively (16% vs 6%, p = 0.004). Prominent themes that emerged from open-ended responses on regrets during the pandemic included missing out on school social or sports events, missing out being with friends, and attending family events or vacations.

**Conclusion:**

In this survey of high school students conducted 2 years after the onset of COVID-19 in the United States, 47% of females and 21% of males reported moderate or severe anxiety symptoms as assessed by the GAD-7. Whether heightened anxiety results in functional deficits is still uncertain, but resources for assessment and treatment should be prioritized.

## Introduction

The long-term impact of the COVID-19 pandemic on the mental health of adolescents is still under investigation. A meta-analysis of 136 studies from various populations affected by COVID-19 found that at least 15–16% of the general population experienced symptoms of anxiety or depression [1]. The Adolescent Behaviors and Experiences Survey (ABES) an online survey of a probability-based nationally representative sample of students in grades 9–12 (N = 7,705) collected from January-June of 2021 in the United States, found that 37% of students experienced poor mental health during the pandemic [2]. During the 12 months before the survey, 44% experienced persistent feelings of sadness or hopelessness, 19.9% had seriously considered attempting suicide, and 9.0% had attempted suicide [2].

Adolescence is a development stage characterized by profound physiological, psychological and social change that could make them particularly vulnerable to stressful events [3, 4]. Although fears of infection, sadness related to loss, and overwhelming uncertainty was experienced by people of all ages, the widespread disruption of education had profound effects on the mental health of children and adolescents [5]. Remote learning, restrictions placed on social gathering, cancellation or modification of sports or clubs, and in-school activities and events present major challenges for the education and social growth of young people. The disruption of school routines and isolation, loss of support from peers and teachers, not only makes learning difficult but can heighten the anxiety that adolescents already feel about their education and career [6]. Even before the pandemic, there were reports of increases in anxiety, depression, substance use among adolescents faced with excessive pressures to excel in affluent settings [7]. Social support from other students and teachers, especially during stressful times, is critical for the social-emotional well-being of adolescents and for sustaining academic engagement and motivation [8–10]. The COVID Experiences Survey, a nationwide survey of 567 adolescents in grades 7–12 performed in 2020, found that adolescents receiving virtual instruction reported more mentally unhealthy days, more persistent symptoms of depression, and a greater likelihood of considering suicide than students in other modes of instruction [11].

The Adolescent Behaviors and Experiences Survey and COVID Experiences Survey both assessed level of stress, symptoms of depression and consideration of suicide among high school students but did not specifically include an evaluation of anxiety [2, 11]. Several smaller published surveys of mental health among adolescent high school students in the United States included assessments of anxiety, although not all of them included validated measures of anxiety or examined the consequences of heightened anxiety [12, 13]. In addition, all were performed in 2020, during the first wave of the infection. To our knowledge, few if any studies have examined longer-term consequences of the COVID-19 pandemic on adolescent anxiety using validated tools. The goal of this study was to evaluate the longer-term impact of the COVID-19 pandemic on generalized anxiety in high school students using the General Anxiety Disorder-7 (GAD-7), a validated self-report measure, at the end of 2021. Variations by gender and the impact of anxiety on achievement goals, future aspirations and outlook of students were also explored.

## Materials and methods

### Study design

This study was conducted at a single public high school in Westchester County of the State of New York. New York was one of the epicenters during the first wave of the COVID-19 epidemic in the United States with a peak daily infection rate of over 9,000 cases/day in April 2020. In response to the New York State Education Department Executive Order, the high school was closed to in-person learning in March 2020 and transitioned to online classes (remote learning). The school remained closed to in-person learning for the remainder of the academic year. After summer break, the school re-opened with remote learning and provided the option for students to return to hybrid learning on October 7, 2020. Hybrid learning consisted of in-person school for half the week and remote learning for the other half of the week with half the capacity of students in the school at any given time. The school also allowed students to continue with full-time remote learning. This decision was made to balance the benefits of in-person learning with safety guidelines by reducing the total number students in school at any given time. On April 7, 2021, the school transitioned from hybrid learning to 100% in-person learning for the remainder of the academic year but still allowed students the option of remote learning. On September 7, 2021, the school re-opened after summer break to 100% in-person learning for all students without the remote learning option. The decision to transition to in-person learning for all students in September 2021 was based upon the low case rates of COVID and the availability of COVID vaccination. The FDA announced the emergency use authorization of the Pfizer-BioNTech COVID-19 vaccine for individuals 16 years of age and older on December 11, 2020 and for individuals 12 years of age and older on April 9, 2021.

### Participants

A total of 521 students were enrolled in the high school, with the following numbers of students in each grade: 142 in 9^th^,130 in 10^th^, 120 in 11^th^ and 128 in 12^th^ grade. The student body composed of 242 females and 279 males, with the following racial/ethnic distribution: 79% White, 13% Asian, 7% Black/African American, 1% American Indian/Native American. This non-selective public high school is the only high school in town. For context, the racial distribution of Westchester County was 73% White, 7% Asian, 17% Black in the 2019 census, with a median household income (in 2019 dollars) of $96,610 and 49% of the population over 25 years having a bachelor’s degree or higher. In the same period, the median household income in the United States was $68,703 with 22.5% of population age 25 and older having bachelor’s degree or higher.

The Irvington School Board approved the survey instruments and the overall study. All students attending the high school in 9^th^-12^th^ grade were eligible to participate. Participation was voluntary, each survey question was optional, and there were no incentives for completion of survey. All participants completed an electronic parental consent and student assent prior to performing the online survey. A survey link was posted by the science teachers on the science classroom pages for all eligible students to complete on November 24, 2021. Science teachers continued to promote the survey until its closure on January 13, 2022.

### Study instruments

The survey was conducted online via Google Forms software (version 2018) in English, and contained 25 questions, 23 of which were multiple choice. Participants took approximately 10–15 minutes to complete the survey. The Generalized Anxiety Disorder-7 scale (GAD-7), a validated 7-item self-administered tool to evaluate anxiety severity, was utilized to measure anxiety [14]. GAD-7 has been utilized in adolescents and demonstrates an acceptable specificity and sensitivity for detecting clinically significant anxiety symptoms in comparison to the Pediatric Anxiety Rating Scale [15]. Participants are asked how often they were bothered by each of the following symptoms during the last 2 weeks with a 4-point scale ranging from “not at all” (0 points) to “nearly every day” (3 points): feeling nervous, anxious or on edge; not being able to stop or control worrying; worrying too much about different things; trouble relaxing; being so restless that it is hard to sit still; becoming easily annoyed or irritable; feeling afraid as if something awful might happen. The total score indicates the level of anxious symptoms ranging from minimal/no anxiety (0–4), mild (5–9), moderate (10–14) and severe (≥15).

Demographic data were collected, including current grade (9–12), gender (female, male, transgender man, transgender woman, non-binary, other), race (American Indian or Alaska Native, Asian, Black or African American, Hispanic or Latino, Native Hawaiian or other Pacific Islander, other), whether attending school by hybrid or remote learning, and COVID-19 vaccination status (none, partial or full series).

Several questions were developed by the study team through an iterative process that included initial development of question by the student researcher, refinement of wording by all investigators including experts in adolescent development and cognition, and testing for comprehension and clarity through review by 2 additional students. Four questions on whether students had more anxiety upon return to in-person learning in April 2021 (after hybrid or remote learning) or September 2021 (after summer break), and factors associated with the anxiety associated with in-person learning were assessed. Thirteen questions were included to assess importance of relationships, safety, achievements and future aspirations (5-point Likert scale from very important to not important): having friends/socializing; perception by friends; making parents proud; maintaining family relationships; good health (not getting COVID); feeling safe; getting good grades; graduating high school; attending college; becoming famous; having adventure; having money/wealth; and having your own family. One additional question addressed outlook on future (5-point Likert scale from strongly agree to strongly disagree): “I think I will have more opportunities in life than my parents.” Three questions designed by the team assessed the impact of COVID-19 (5-point Likert scale from strongly agree to strongly disagree): “The COVID-19 pandemic has changed me significantly”; “The COVID-19 pandemic has made me mature faster”; Overall, the COVID-19 pandemic has affected my personal growth negatively.”

Two additional open-ended questions were included to allow students to reflect upon opportunities lost and gratitude experienced during COVID-19: “Share one moment that you regret missing out on during the COVID-19 pandemic,” and “Share one moment when you felt grateful during the COVID-19 pandemic”

### Data analysis

#### Quantitative analysis

Overall frequencies for demographics, GAD-7, and responses to questions on the importance of relationships, safety, achievements and opportunities were examined. Bivariate analyses by demographics characteristics (gender, grade, and learning type) were conducted with each response. Chi-square tests were conducted to determine whether responses differed by gender, grade, learning type, and severity of anxiety. All analyses were performed using SPSS Statistics for Mac, version 28.0 (SPSS Inc, Chicago, Ill, USA).

#### Qualitative analysis

The answers to each open-ended question were evaluated for themes. The iterative process took the form of a data analysis spiral such that following data collection, the data was organized, read and notated for emerging ideas, described and classified by thematic codes, assessed and interpreted, and presented in this research report [16]. Author 1 read all the responses and compiled the data and created preliminary thematic codes. Author 2 reviewed the thematic codes and believed that thematic saturation had been reached. Author 1 then discussed all preliminary codes with all authors who provided additional memos. Representative excerpts for each theme are presented in Table 4. Data saturation was defined using the grounded theory standpoint by Urquhart, that defined saturation as “the point in coding when you find that no new codes occur in the data. There are mounting instances of the same codes, but no new ones”[17, 18].

## Results

Among the 546 students enrolled in the high school, 155/546 (28%) completed the survey, including 90 females, 59 males, and 6 students who did not identify as gender binary. Since the number of gender non-binary students was too small to include as a separate group in analyses looking at gender differences, results were presented only for students who self-identified as either female or male (n = 149) (**Table 1**). The proportion of respondents was greater among females (90/262, 34%) than males (59/284, 21%). The response rates were much lower in 12^th^ grade (25/137, 18%) than in 9^th^ grade (61/139, 44%). The students were mostly White (69%), Asian (16%) or multi-racial (9%), predominantly engaged in hybrid learning (86%), and almost all (97%) fully or partially vaccinated against SARS-CoV-2 at the time of survey completion (**Table 1**).

**Table 1:** Demographics, vaccination status, and anxiety related to in-person learning, stratified by gender.

Variable	All (n = 149)*	Females (n = 90)	Males (n = 59)	P value
**Learning type**				
Hybrid	128 (86%)	79 (88%)	49 (83%)	0.45
Remote	21(14%)	11 (12%)	10 (17%)	
**Grade**				
9	59 (40%)	28 (31%)	31 (53%)	0.05
10	26 (17%)	16 (18%)	10 (17%)	
11	40 (27%)	28 (31%)	12 (20%)	
12	24 (16%)	18 (20%)	6 (10%)	
**Race**				
White	103 (69%)	67 (74%)	36 (61%)	0.12
Asian	24 (16%)	11 (12%)	13 (22%)	
Black	3 (2%)	2 (2%)	1 (2%)	
Hispanic	4 (3%)	1 (1%)	3 (5%)	
Multiracial	13 (9%)	9 (10%)	4 (7%)	
NA	2 (1%)		2 (3%)	
**Vaccination status**				
None	5 (3%)	2 (1%)	3 (2%)	0.24
Partial	3 (2%)	3 (2%)	0 (0%)	
Full series	141 (95%)	85 (94%)	56 (95%)	
**I felt anxious returning to 100% in-person learning in April 2021.**				
Yes	63 (42%)	47 (52%)	16 (27%)	0.002
No	79 (53%)	39 (43%)	40 (68%)	
NA	7 (5%)	4 (4%)	3 (5%)	
**Why did you feel anxious returning to in-person learning in April 2021?**				
COVID	32 (21%)	23 (26%)	9 (15%)	0.024
Schoolwork	12	9 (10%)	3 (5%)	
Social interactions	23 (15%)	18 (20%)	5 (8%)	
No anxiety	74 (50%)	35 (39%)	39 (66%)	
NA	5 (5%)	3 (5%)	9 (5%)	
**I felt less anxious returning to in-person learning in September 2021, than in April 2021**				
Yes	72 (48%)	46 (51%)	26 (44%)	0.47
No	74 (50%)	43 (48%)	31 (53%)	
NA	3 (2%)	1 (1%)	2 (3%)	
**Why did you feel less anxious returning to in-person learning in September 2021?**				
COVID vaccine	56 (38%)	36 (40%)	20 (34%)	0.73
Less COVID cases	2 (1%)	1 (1%)	1 (2%)	
School safety measures	2 (1%)	2 (2%)	0 (0%)	
Normalization of social interactions	24 (16%)	13 (14%)	11 (18%)	
More anxious in September than April	14 (9%)	10 (11%)	4 (7%)	
No anxiety	39 (26%)	21 (23%)	18 (30%)	

* Non-binary (n = 6) students were not included stratified analysis for gender

Overall, 54/149 (36%) of the students had GAD-7 scores in the range for moderate or severe anxiety (scores≥10), with a greater proportion of the females than males experiencing moderate/severe anxiety (47% vs 21%, X^2^ = 21.3984, P<0.001) (**Table 2**). Among students who answered yes to any of the GAD-7 questions, 3% reported that anxiety made it extremely difficult and 12% reported that anxiety made it very difficult to do their work, take care of things at home, or get along with other people. More females than males (19% vs 7%, p<0.01) reported that anxiety made it very or extremely difficult to do their work, take care of things at home, or get along with other people (**Table 2**). Severity of anxiety did not differ between students in the lower (9^th^ and 10^th^) versus the upper (11^th^ and 12^th^) grades. Severity of anxiety also did not differ between students engaged in hybrid versus remote learning (**Table 2**).

**Table 2:** Severity of anxiety assessed by GAD-7 stratified by gender, grade and learning type.

Variable	All (n = 149)*	Female (n = 90)	Male (n = 59)	P value	Grades 9 &10 (n = 88)	Grades 10 & 11 (n = 67)	P value	Hybrid learning (n = 130)	Remote learning (n = 20)	P value
**GAD-7**										
Minimal Anxiety (0–4)	49 (33%)	17 (19%)	32 (54%)	<0.001	33 (36%)	16 (24%)	0.11	42 (31%)	8 (40%)	0.59
Mild Anxiety (5–9)	46 (31%)	31 (34%)	15 (25%)		25 (28%)	23 (34%)		40 (32%)	6 (31%)	
Moderate Anxiety (10–14)	34 (23%)	27 (30%)	7 (12%)		15 (17%)	20 (30%)		29 (22%)	2 (10%)	
Severe Anxiety (15–21)	20 (13%)	15 (17%)	5 (9%)		15 (17%)	8 (12%)		19 (15%)	4 (20%)	
**How difficult have the anxiety symptoms made it for you to do your work, take care of things at home, or get along with other people?**										
Somewhat difficult	88 (60%)	58 (65%)	30 (53%)	<0.01						
Very difficult	17(12%)	13 (15%)	4 (7%)							
Extremely difficult	5 (3%)	4 (4%)	1 (2%)							

* Non-binary (n = 6) students were not included stratified analysis for gender

More females than males felt anxious returning to in-person school in April 2021 (52% vs 27%; X^2^ = 9.3457, p = 0.002) (**Table 1**). COVID-19 vaccinations were available for individuals 16 years of age or older by December 2020 with emergency use authorization for individuals 12 years of age and older only granted on April 9, 2021. All of the major factors contributing to anxiety measured were more frequently reported in females than males: fear of getting COVID-19 (26% vs 15%), anxiety toward social interactions (20% vs 8%), and schoolwork (10% vs 5%). By September 2021, 51% of females and 44% of males reported feeling less anxious for in-person school than in April 2021. The primary reasons reported for decreased anxiety were the receipt of COVID-19 vaccinations (38%) and normalization of social interactions with in-person school (16%) (**Table 1**).

Overall, 34% of students strongly agreed that the COVID-19 pandemic “changed me significantly” and 24% strongly agreed that it “made me mature faster” (**Table 3A**). However, only 8% of students strongly agreed that the COVID-19 pandemic “has affected my personal growth negatively.” More females reported that COVID-19 affected their personal growth negatively, but it did not reach statistical significance (11% vs 5%, p = 0.15). In comparison to students with either mild anxiety or no anxiety (GAD-7<10), students with moderate to severe anxiety (GAD-7≥10) were more likely than students with either mild anxiety or no anxiety (GAD-7<10) to strongly agree that the COVID-19 pandemic changed them significantly (51% vs 28%, p = 0.05), made them mature faster (44% vs 16%, p = 0.004), and affected their personal growth negatively (16% vs 6%, p = 0.004) (**Table 3B**).

**Table 3:** Impact of COVID-19 stratified by gender (3a) and by severity of anxiety by GAD-7 (3b).

**Table 3a**	**All (n = 147)**	**Females (n = 88)**	**Males (n = 59)**	**P value**
**The COVID-19 pandemic has changed me significantly**				
Strongly agree	51 (34%)	34 (38%)	17 (29%)	0.58
Somewhat agree	41 (28%)	24 (27%)	17 (29%)	
Neither agree or disagree	33 (22%)	19 (21%)	14 (24%)	
Somewhat disagree	17 (12%)	10 (11%)	7 (12%)	
Strongly disagree	6 (4%)	2 (2%)	4 (7%)	
**The COVID-19 pandemic has made me mature faster**				
Strongly agree	36 (24%)	26 (30%)	10 (17%)	0.20
Somewhat agree	46 (31%)	22 (25%)	24 (41%)	
Neither agree or disagree	41 (28%)	24(27%)	17 (29%)	
Somewhat disagree	19 (13%)	12 (14%)	7 (12%)	
Strongly disagree	5 (3%)	4 (5%)	1 (2%)	
**Overall, the COVID-19 pandemic has affected my personal growth negatively**				
Strongly agree	13 (8%)	10 (11%)	3 (5%)	0.15
Somewhat agree	28 (19%)	21 (24%)	7 (12%)	
Neither agree or disagree	31 (21%)	17(19%)	14 (24%)	
Somewhat disagree	46 (31%)	27 (30%)	19(32%)	
Strongly disagree	30 (20%)	14 (16%)	16 (27%)	
**Table 3b**	**All (n = 148)**	**GAD-7<10 (None-mild anxiety) (n = 105)**	**GAD-7≥10 (Mod-severe anxiety) (n = 43)**	**P value**
**The COVID-19 pandemic has changed me significantly**				
Strongly agree	51(34%)	29(28%)	22 (51%)	0.05
Somewhat agree	41 (28%)	33 (31%)	8 (19%)	
Neither agree or disagree	33 (22%)	23 (22%)	10 (23%)	
Somewhat disagree	17 (12%)	15 (14%)	2 (8%)	
Strongly disagree	6 (4%)	5 (5%)	1 (2%)	
**The COVID-19 pandemic has made me mature faster**				
Strongly agree	36 (24%)	17 (16%)	19 (44%)	0.004
Somewhat agree	46 (31%)	37 (36%)	9 (21%)	
Neither agree or disagree	41 (28%)	34 (32%)	7 (16%)	
Somewhat disagree	19 (13%)	12 (12%)	7 (16%)	
Strongly disagree	5 (3%)	4 (4%)	1 (2%)	
**Overall, the COVID-19 pandemic has affected my personal growth negatively**				
Strongly agree	13 (9%)	6 (6%)	7 (16%)	0.004
Somewhat agree	28 (19%)	15 (14%)	13 (30%)	
Neither agree or disagree	31 (21%)	20 (19%)	11 (26%)	
Somewhat disagree	46 (31%)	38 (36%)	8 (19%)	
Strongly disagree	30 (20%)	26 (25%)	4 (9%)	

We further explored whether moderate/severe anxiety affected students’ outlook on relationships, safety, achievements, aspirations and opportunities. Over half of students reported the following life factors as very important: having friends/socializing (53%), maintaining good health and not getting COVID-19 (53%), getting good grades (62%), graduating high school (82%), and attending college (74%) (**Table 4**). Females were more likely than males to regard the following factors as very important: money/wealth (28% vs 12%, p<0.01) and having your own family (39% vs 29%, p = 0.02), but did not differ from boys in other reported factors. Students with moderate to severe anxiety (GAD-7≥10) were more likely than students with mild or no anxiety to regard the following as very important: attending college (81% vs 70%, p = 0.04), becoming famous (9% vs 1%, p = 0.04), and having your own family (44% vs 31%, p = 0.01). Only 23% of students reported that they strongly agree with the statement “I will have more opportunities in my life than my parents”, without apparent differences by anxiety status (**Table 4**).

**Table 4:** Importance of relationships, safety, achievements, aspiration and opportunities stratified by anxiety level.

Variable	All (n = 148)	GAD-7<10 (None-mild anxiety) (n = 105)	GAD-7≥10 (Mod-severe anxiety) (n = 43)	P value
**Relationships**				
**Having Friends/Socializing**				
Very important	78 (53%)	51 (49%)	27 (63%)	0.21
Somewhat important	38 (26%)	31 (30%)	7 (16%)	
Neutral	21 (14%)	13 (13%)	8 (19%)	
Not very important	9 (6%)	8 (8%)	1 (2%)	
Not important	1 (1%)	1 (1%)	0 (0%)	
**How my friends perceive me**				
Very important	22 (15%)	12 (12%)	10 (23%)	0.12
Somewhat important	44 (30%)	28 (27%)	16 (37%)	
Neutral	39 (27%)	29 (28%)	10 (23%)	
Not very important	35 (24%)	29 (28%)	6 (14%)	
Not important	7 (5%)	6 (6%)	1 (2%)	
**Making parents proud**				
Very important	61 (42%)	37 (36%)	24 (56%)	0.06
Somewhat important	49 (34%)	39 (38%)	10 (23%)	
Neutral	25 (17%)	21 (20%)	4 (9%)	
Not very important	8 (6%)	5 (5%)	3 (7%)	
Not important	3 (2%)	1(1%)	2 (5%)	
**Maintaining family relationships**				
Very important	60 (41%)	3 (40%)	25 (43%)	0.08
Somewhat important	41 (28%)	23 (26%)	18 (31%)	
Neutral	30 (21%)	18 (18%)	12 (28%)	
Not very important	13 (9%)	9 (9%)	4 (9%)	
Not important	2 (1%)	0 (0%)	2 (5%)	
**Safety**				
**Good health (not getting COVID-19)**				
Very important	78 (53%)	54 (52%)	24 (56%)	0.38
Somewhat important	29 (20%)	22 (21%)	7 (16%)	
Neutral	28 (21%)	22 (14%)	6 (19%)	
Not very important	7 (5%)	3 (3%)	4 (9%)	
Not important	5 (3%)	3 (3%)	2 (5%)	
**Feeling safe**				
Very important	66 (45%)	43 (42%)	23 (54%)	0.31
Somewhat important	34 (23%)	27 (27%)	7 (16%)	
Neutral	27 (19%)	17 (17%)	10 (23%)	
Not very important	15 (10%)	13 (13%)	2 (5%)	
Not important	3 (2%)	2 (2%)	1 (2%)	
**Achievements and Aspirations**				
**Getting good grades**				
Very important	91 (62%)	66 (64%)	25 (58%)	0.58
Somewhat important	37 (25%)	25 (24%)	12 (28%)	
Neutral	12 (8%)	7 (7%)	5 (12%)	
Not very important	7 (5%)	6 (6%)	1 (2%)	
Not important	0 (0%)	0 (0%)	0 (0%)	
**Graduating High School**				
Very important	120 (82%)	83 (80%)	37 (86%)	0.13
Somewhat important	14 (10%)	13 (13%)	1 (2%)	
Neutral	11 (8%)	6 (6%)	5 (12%)	
Not very important	2 (1%)	2 (2%)	0 (0%)	
Not important	0 (0%)	0 (0%)	0 (0%)	
**Attending college**				
Very important	108 (74%)	73 (70%)	35 (81%)	0.04
Somewhat important	20 (14%)	18 (17%)	2 (5%)	
Neutral	13 (9%)	7 (7%)	6 (14%)	
Not very important	6 (4%)	6 (6%)	0 (0%)	
Not important	0 (0%)	0 (0%)	0 (0%)	
**Becoming famous**				
Very important	5 (3%)	1 (1%)	4 (9%)	0.04
Somewhat important	11 (8%)	9 (9%)	2 (5%)	
Neutral	26 (18%)	15 (14%)	11 (26%)	
Not very important	45 (31%)	33 (32%)	12 (28%)	
Not important	60 (41%)	46 (44%)	14 (33%)	
**Having adventure**				
Very important	29 (20%)	16 (16%)	13 (30%)	0.10
Somewhat important	43 (30%)	30 (29%)	13 (30%)	
Neutral	39 (27%)	27 (26%)	12 (28%)	
Not very important	32 (22%)	28 (27%)	4 (9%)	
Not important	3 (2%)	2 (2%)	1 (2%)	
**Having money/wealth**				
Very important	32 (22%)	19 (18%)	13(30%)	0.20
Somewhat important	52 (36%)	39 (38%)	13 (30%)	
Neutral	45 (31%)	32 (31%)	13 (30%)	
Not very important	14 (10%)	12 (12%)	2 (5%)	
Not important	3 (2%)	1 (1%)	2 (5%)	
**Having your own family**				
Very important	51 (35%)	32 (31%)	19 (44%)	0.01
Somewhat important	37 (25%)	32 (31%)	5 (12%)	
Neutral	27 (19%)	13 (14%)	13 (30%)	
Not very important	22 (15%)	19 (18%)	3 (7%)	
Not important	9 (6%)	6 (4%)	3 (2%)	
**Opportunity**				
**I think I will have more opportunities in life than my parents**				
Strongly agree	33 (23%)	10 (22%)	23 (24%)	0.17
Somewhat agree	46 (32%)	36 (35%)	10 (24%)	
Neither agree or disagree	56 (39%)	39 (38%)	17 (41%)	
Somewhat disagree	8 (6%)	5 (5%)	3 (7%)	
Strongly disagree	2 (1)	0 (0%)	2 (5%)	

In response to “share one moment that you regret missing out on during COVID-19 pandemic,” the following themes emerged, from most common to least common: missing out on school social events and sports; being with friends; family events and vacations, wasted new opportunities that were presented during COVID-19 pandemic, and celebrating milestones like bar mitzvahs, sweet-sixteens and birthdays. In response to “share one moment when you felt grateful during the COVID-19 pandemic,” the following themes emerged, from most common to least common: connecting with friends and family, health and safety, having time for personal development, moments during which there was a sense of return to normalcy, and the decreased stress of remote learning (**Table 5**). Generally, the noted themes were similar in students with moderate-severe anxiety versus those with mild or no anxiety. However, in comparison to students with mild or no anxiety, more students with moderate-severe anxiety expressed that they regret missing out on being with friends, and less expressed regret for missing out on school-related social events such as the prom, school trips, or sports competitions. Notably, while all the students with moderate-severe anxiety reported missing out on something, 5% of students with either mild or no anxiety reported that they did not miss out on anything during the COVID-19 pandemic. Also, more students with moderate-severe anxiety expressed that they were grateful for health and safety and situations that provided a sense of normalcy.

**Table 5:** Examples of moments of regret and gratefulness.

Themes	Examples	
	Moderate-Severe Anxiety (GAD-7≥10)	Minimal to No Anxiety (GAD-7<10)
**Share one moment that you regret missing out on during the COVID-19 pandemic**		
**School social or sports event**	**29% of responses** “My state competition was cancelled after I qualified” “I regret missing out on opportunities in sports since I was unable to join certain teams for the sports I play due to the pandemic.” “PROM”	**43% of responses** “I feel like we missed out on basic high school social interactions, i just regret feeling so isolated” “definitely on my second swim season ever, and all of the activity I missed” “I regret missing out on trips I would have taken, both for school and recreationally”
**Being with friends**	**26% of responses** “Being able to hang out with friends easily and not being able to travel as much.” “Getting to have more social interactions with friends.” “Lots of friend hangouts”	**12% of responses** “I regret missing out on a lot of social interaction with friends, since I couldn’t talk to them in-person for more than a year.” “Missing out on social interactions/leaving the house” “For 4 months, I couldn’t hang out with my significant other unless we were outside, 6 feet apart, and had masks on and I felt like I missed out on that connection that could have been made between us much stronger”
**Family events and vacations**	**16% of responses** “Christmas. I unfortunately got Covid during Christmas so I had to miss out on family traditions.” “Family reunion in August 2020 was cancelled” “Going to Korea to visit my family and other vacations”	**14% of responses** “I was not able to visit my grandmother. We had a plan to visit my grandmother in the summer to help her out as much as possible due to growing pain in her legs. Unfortunately, she passed away due to COVID-19 before we could ever get to her.” “I had a trip planned to Israel that I was not able to attend” “I regret missing out on vacations with the rest of my family”
**Missed opportunities for growth**	**16% of responses** “Going outside and cooking. I definitely feel I could of learned a new language and done other extracurriculars outside of school remotely instead of watching tv shows and playing video games because I had the chance.” “I wish that I got to be a CIT at my summer camp and go hike the Grand Canyon”	**16% of responses** “I regret how I pushed back on my studying whereas I could have been using the free time I had to accomplish various tasks and bettering myself for the future.” “I did not take the opportunity to stop procrastinating with all the home time or to pick up many new hobbies.”
**Milestone celebrations**	**8% of responses** “Not being able to hang out with my friends or have my sweet 15 or 16” “Bar/Bat Mitzvahs, because they got cancelled” “my birthday”	**5% of responses** “I regret missing out on some of the bat and bar mitzvahs of my peers and friends.” “locked inside for my fifteenth and sixteenth birthday on COVID related grounds.”
**Share one moment when you felt grateful during the COVID-19 pandemic**		
**Connecting with friends and family**	**32% of responses** “One moment when I felt grateful during the COVID-19 pandemic was when I got to spend more time with my mom, brother, and dogs at home. It forced us to do more together because we didn’t have anything else to do.” “The ability to bond with my sister during quarantine. I also fostered kittens while I sat at home which made it more bearable.” “Going for walks with my best friend”	**45% of responses** “I felt grateful when I was able to connect with some of my friends on a deeper level. Without the pandemic, I don’t think I would have made the friendships I have today” “I got to spend more time with my mom, brother, and dogs at home. It forced us to do more together because we didn’t have anything else to do.” “Thankful for FaceTime when I wasn’t able to see my friends in person” “When I got my dog during quarantine”
**Health and Safety**	**32% of responses** “I felt grateful when I was finally able to hug my grandparents since they were very cautious during the pandemic.” “During quarantine in 2020- grateful I didn’t live in an abuse [sic] household” “I felt grateful because I never got COVID-19. However, specifically I was very grateful when my grandma bounced back and is ok now after she was extremely sick.” “When my dad brought me to get the vaccine”	**23% of responses** “By the end of the pandemic, no one in close relation to me had passed.” “I had my own house and none of my family was sick.” “When I got a mild case of covid and had no bad symptoms” “For food and shelter and a stable income” “I felt grateful that I could take the COVID-19 vaccine as early as possible. In Bangladesh, my home country, most of the population still do not have access to COVID vaccines.”
**Time for personal growth**	**8% of responses** “When I didn’t have to attend school in person and I could do stuff at home” “Not having to be in school completely and being able to find” new fashion and drawing ideas. I have some more motivational stress.”	**10% of responses** “I was able to take my time and think more.” “When I had more free time to do stuff I like” “It gave me time to slow down and it taught me that sometimes being alone is good”
**Sense of normalcy**	**13% of responses** “When I got to go to the beach for my birthday Christmas” “The first time I got in a car with my best friend after she got her license.”	**7% of responses** “I was grateful during my older brother’s graduation parade still happening. I was grateful that the pandemic didn’t completely ruin his senior year.” “When I had my bar mitzvah on my street”
**Decreased stress of remote learning**	**13% of responses** “I felt grateful because I could have the option to relax and rest after school and be able to develop a clear mindset.” “I liked waking up later for school and staying home”	**16% of responses** “I was grateful that there wasn’t as much schoolwork and tests”. “school was a lot easier” “When my grades went up because of the grading system during the pandemic.”

## Discussion

In one of the first reports on levels of anxiety in high school students during the second year of the COVID pandemic, this study found that 36% of the students reported moderate or severe anxiety, disproportionately affecting females. Although the GAD-7 is a screener for anxiety and meant to over detect, anxiety scores in this range are considered clinically meaningful and indications for further assessment and/or referral to a mental health professional for more definitive diagnoses. These surveys were completed in late 2021 at a point when over 95% of students had received partial or full vaccinations; therefore, our data suggest that the impact of COVID-19 on the generalized anxiety of high school students may be long-lasting.

Our findings are consistent with several large mental health surveys that included measures of anxiety were conducted on university students in 2020, earlier in the COVID-19 pandemic, and found that females were more likely to report moderate to severe general anxiety then males. The Healthy Minds Survey 2020, one of the largest studies of university students in the United States (N = 36,875), found that 32.2% of students reported moderate to severe anxiety, with a higher proportion in females than males (66.6% vs 28.6% of males) [19]. Similarly, a survey of over 69,000 university students in France found that females were more likely to report high levels of anxiety than males (30.8% vs 17.1%) [20].

As noted previously, the largest mental health surveys conducted among high school students in the United States did not specifically include an evaluation of anxiety [2, 11], but anxiety was included in two smaller studies. Gazmararian et al. surveyed racial/ethnically and socioeconomically diverse students at 2 semi-rural public high schools in Georgia in 2020 and found that 25% of students were worried about the COVID-19 pandemic and a negative financial impact, with a similar gender difference in girls versus boys (29% vs 16%, p<0.0001) [13]. The Policy and Communication Evaluation (PACE) Vermont is an online cohort study of 212 adolescents (ages 12–17) and 662 young adults (ages 18–25) that completed questionnaires in the Fall of 2019 and 2020, before and after the onset of the COVID-19 pandemic [12]. The prevalence of anxiety symptoms measured by the GAD-2 increased from 24.3% to 28.4% among adolescents after COVID-19, similar to the increase from 35.3% to 42.3% observed among young adults [12].

In our study, 36% of high school students had moderate/severe anxiety by GAD-7, which is slightly higher than the prevalence in aforementioned high school studies, and similar to the prevalence among college students in the Healthy Minds Survey. Female high school students were more likely to report moderate or severe anxiety. Importantly, this study explored potential reasons for anxiety upon return to in-person learning in April 2021, informed by high school students (including lead author) and a greater proportion of females than males endorsed each category: COVID-19 (26% vs 15%), schoolwork (10% vs 5%) and social interactions (20% vs 8%). These data suggest that female high school students had higher anxiety levels not only because of fear of COVID, but also because of more normative stressors pre-COVID, such as school and social pressures. Furthermore, females reported more negative effects of their anxiety compared to boys, with 19% reporting that it is “extremely difficult to do their work, take care of things at home, or get along with other people” as compared to only 9% of males. Notably, severity of anxiety did not appear to differ between students in the lower (9^th^ and 10^th^) versus the upper (11^th^ and 12^th^) grades. This was unexpected given higher levels of stress associated with standardized testing and college applications in the upper grades. Severity of anxiety also did not differ between students engaged in hybrid versus remote learning (**Table 2**). However, since most of the students were engaged in hybrid learning (87%), our power to detect differences was limited. Other investigators found no difference in risk for anxiety among students with remote versus in-person education [21]; however, the role of hybrid learning has never been adequately assessed.

Students who reported moderate/severe anxiety had very different responses than students with either mild or no anxiety regarding the impact of the COVID-19 pandemic. Students with moderate/severe anxiety were far more likely to strongly agree that the COVID pandemic changed them (51% vs 28%), made them mature faster (44% vs 16%), and affected their personal growth negatively (16% vs 6%). It is possible that COVID-19 had a greater negative impact on these students resulting in higher anxiety levels, or that students with higher anxiety levels before the pandemic were more susceptible to the negative effects of COVID-19. This question cannot be addressed without pre-pandemic data on these students. However, it is interesting that even though students with moderate/severe anxiety perceived a greater negative impact of COVID-19, they did not differ from other students in their hopes and aspirations for the future. In fact, more students with moderate to severe anxiety responded that attending college, becoming famous, and having their own family was very important (**Table 4**). This may also reflect a greater underlying expectation for success and a desire for safety and security among students with greater anxiety. This is an important area for future study. While students reported being concerned about good health and “not getting COVID-19,” less than half of the students (45%) rated “feeling safe” as very important. While these data may reflect the higher risk tolerance of adolescents in general vs other age groups, the data also suggest that the heightened awareness of safety measures for COVID-19 did not translate into generalized fear affecting other aspects of their lives. Overall, these data suggest that despite the relatively high proportion of students reporting anxiety, the majority did not perceive negative effects and thus appeared to be coping with the stressors of COVID-19.

This study was not designed for formal qualitative research, but there were two open-ended questions on regrets and gratitude. Missing out on school social or sports events was the most common theme, followed by missing out being with friends or attending family events or vacations. Several students also articulated missed opportunities for growth presented by COVID-19 and shared regrets for not accomplishing more with the extra time. Students shared their gratitude mostly for connecting with friends and family and for health and safety. There were also appreciations written for having a time for personal growth, moments during COVID-19 that provided a sense of normalcy, and the decreased stress from school that remote learning offered (**Table 4**). Based upon exploratory analyses, it appeared that students with moderate-severe anxiety were more likely to regret missing out on being with friends, less likely to regret missing out on school social or sports events, and more likely to be grateful for health and safety. Further work could examine how these constructs may be important for adolescents experiencing moderate-severe anxiety.

There are now several longitudinal studies of change in mental health measures among children and young adults before and during the COVID-19 pandemic [22]. Several comprehensive studies of college and university students in the United States include data on pre-pandemic mental health, analyses of predictors, and a focus on serious psychiatric and alcohol/drug use outcomes [23, 24], but data are lacking for high school students. Stamatis et al found that the disruption due to the pandemic and limited confidence in the government response were the main predictors of depression among college students [24]. Bountress et al found that COVID-19 worry predicted post-traumatic stress disorder (PTSD), depression and anxiety even after adjusting for pre-pandemic symptom levels [23]. In addition, housing/food concerns predicted PTSD, anxiety and depression symptoms as well as suicidal ideation, after adjusting for pre-pandemic symptoms in college students [23]. Comprehensive longitudinal studies are necessary to assess the true impact of COVID on mental health in high school students. In particular, studies should assess whether symptoms are associated with serious clinical outcomes such as suicidal ideation, alcohol and substance misuse and missed milestones such as graduation from high school, admission to college, and employment.

### Strengths and limitations

A strength of our study was the use of the well validated and extensively used GAD-7 to measure anxiety symptoms. There were no data on anxiety for the students prior to COVID-19 as a baseline for comparison nor measures of other indicator of mental health such as depression and suicidality. Other limitations of this study include the performance of the survey at a single high school—our sample size was limited and the analyses were performed on a convenience sample. While only 28% of the study body responded to the survey, this response rate was similar to the response rates of other high school surveys performed in the United States [12, 13, 25]. The lack of racial/ethnic diversity in the student population also limits generalizability to other populations of adolescents. We did not include potential risk factors elicited in other studies such as prior psychiatric history, financial hardship, or illness in family in our survey. We were also unable to evaluate the impact of hybrid versus remote learning on anxiety, since very few of our students chose remote learning. Lastly, the survey questions we created were done so because nothing specifically existed for this age group, the newness of COVID, and the need to implement questions quickly; therefore, we did not utilize a formal validation process.

## Conclusion

In this survey of high school students performed almost 2 years after the onset of COVID-19 in the United States, a relatively high proportion reported moderate or severe anxiety symptoms as assessed by the GAD-7. Our data suggest that the negative impact of COVID-19 on the anxiety levels of high school students may be long-lasting. Whether the heightened anxiety results in functional deficits is still uncertain, but resources for assessment and treatment should be prioritized.

## Supporting information

S1 Dataset (XLSX)
